# New Appliances and Things Medical

**Published:** 1899-05-20

**Authors:** 


### NEW APPLIANCES AND THINGS MEDICAL.

[We shall be glad to receive, at our Office, 28 & 29, Southampton Street, Strand, London, W.C., from the manufacturers, specimens of all new preparations and appliances which may be brought out from time to time.]

### GYROL REVULSIVE PENCIL.

(Mertens, 64, Holborn Viaduct.)

The application of this pencil, which is the invention of M. Coirre, may in many cases supersede other methods of rubefaction. It has a basis of grease, in which is incorporated a certain proportion of capsicin. When the pencil is rubbed on the skin the grease adheres to it, and the contained capsicin acts as a constant and efficacious rubefacient. The desired effect is immediate and lasting. There is no staining of the skin as with iodine, and no blistering as with croton oil, mustard leaf, and other escharotics, yet its revulsive action is considerable. For counter irritation in inflammations, neuralgias, and other conditions this new method with the gyrol pencil certainly deserves attention. We have tried it with good effect in cases of laryngitis by smearing the grease on the anterior aspect of the neck. One of the chief advantages of this method is that the practitioner can carry in his pocket an effective and immediate rubefacient which can be applied without any preliminary preparation or delay.

### MERCOLEATE SOAP.

(Price's Patent Candle Company, Limited, London, Liverpool, and Manchester.)

This soap contains 5 per cent. of freshly precipitated oleate of mercury. It is one of a series of other medicated soaps prepared by this firm. It should be of value in the treatment of those skin diseases in which a mercurial ointment can be less conveniently applied; or, of course, it may be employed in combination with other applications.

### NEW PROTECTIVE PACKING.

(Horrocks and Co., Standish, Wigan.)

This new protective packing is an improved form of what is generally known as "corrugated paper." It differs from the usual form in that the paper is far more elastic and more readily takes the shape of articles round which it is wrapped than any variety previously known. The paper itself is comparatively thin, but strength and resistance are afforded by a system of double corrugation of mushroom shape. No ordinary amount of pressure can destroy the corrugation or affect the resiliency. By the invention of special machinery the protecting packing can be turned out at a very cheap rate. For chemists and others who send large numbers of bottles by post the new invention should be a boon and a great pecuniary saving.

### L'EAU D'OREZZA (CORSICA).

(London Agency: 6, St. Helen's Place, E.C.)

This water has been known to the foreign members of the medical profession for many years, but it has only recently been introduced to this country. The water referred to issues from a spring at Orezza, in the island of Corsica, at an elevation of 1,968 feet. It is an alkaline, muriated water containing carbonate and protoxide of iron with traces of manganese and arsenious acid; and, further, there is sufficient free carbonic acid to render it slightly effervescent when poured in a tumbler. Its analysis shows it to be an excellent natural ferruginous water, especially indicated in cases of anæmia and chlorosis, where iron in a crude form cannot be tolerated. The presence of traces of manganese and arsenic should render it all the more efficacious in these cases.

